# Increasing SARS-CoV-2 seroprevalence among UK pediatric patients on dialysis and kidney transplantation between January 2020 and August 2021

**DOI:** 10.1007/s00467-023-05983-1

**Published:** 2023-06-01

**Authors:** Holly N. Bamber, Jon Jin Kim, Ben C. Reynolds, Javairiya Afzaal, Andrew J. Lunn, Patrick J. Tighe, William L. Irving, Alexander W. Tarr

**Affiliations:** 1 School of Life Sciences, University of Nottingham, Nottingham, UK; 2 Department of Paediatric Nephrology, Nottingham University Hospitals, Nottingham, UK; 3 Centre for Kidney Research and Innovation, University of Nottingham, Nottingham, UK; 4 Department of Paediatric Nephrology, Royal Hospital for Children, Glasgow, UK; 5 NIHR Nottingham Biomedical Research Centre, Nottingham University Hospitals NHS Trust and the University of Nottingham, Nottingham, UK; 6 Wolfson Centre for Global Virus Research, The University of Nottingham, Nottingham, UK; 7 Microbiology, Queen’s Medical Centre, Nottingham, NG7 2UH UK

**Keywords:** Antibodies, Dialysis, Child, Kidney replacement therapy, SARS-CoV-2, Seroprevalence

## Abstract

**Background:**

Coronavirus disease 2019 (COVID-19) was officially declared a pandemic by the World Health Organisation (WHO) on 11 March 2020, as severe acute respiratory syndrome coronavirus 2 (SARS-CoV-2) spread rapidly across the world. We investigated the seroprevalence of anti-SARS-CoV-2 antibodies in pediatric patients on dialysis or kidney transplantation in the UK.

**Methods:**

Excess sera samples were obtained prospectively during outpatient visits or haemodialysis sessions and analysed using a custom immunoassay calibrated with population age-matched healthy controls. Two large pediatric centres contributed samples.

**Results:**

In total, 520 sera from 145 patients (16 peritoneal dialysis, 16 haemodialysis, 113 transplantation) were analysed cross-sectionally from January 2020 until August 2021. No anti-SARS-CoV-2 antibody positive samples were detected in 2020 when lockdown and enhanced social distancing measures were enacted. Thereafter, the proportion of positive samples increased from 5% (January 2021) to 32% (August 2021) following the emergence of the Alpha variant. Taking all patients, 32/145 (22%) were seropositive, including 8/32 (25%) with prior laboratory-confirmed SARS-CoV-2 infection and 12/32 (38%) post-vaccination (one of whom was also infected after vaccination). The remaining 13 (41%) seropositive patients had no known stimulus, representing subclinical cases. Antibody binding signals were comparable across patient ages and dialysis versus transplantation and highest against full-length spike protein versus spike subunit-1 and nucleocapsid protein.

**Conclusions:**

Anti-SARS-CoV-2 seroprevalence was low in 2020 and increased in early 2021. Serological surveillance complements nucleic acid detection and antigen testing to build a greater picture of the epidemiology of COVID-19 and is therefore important to guide public health responses.

**Graphical abstract:**

**Figure:**
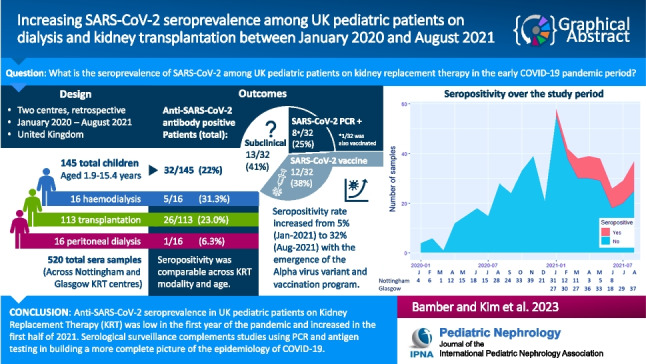
A higher resolution version of the Graphical abstract is available as Supplementary information

**Supplementary Information:**

The online version contains supplementary material available at 10.1007/s00467-023-05983-1.

## Introduction

Coronavirus disease 2019 (COVID-19) was officially declared a pandemic by the World Health Organisation (WHO) on 11 March 2020 as the novel severe acute respiratory syndrome coronavirus 2 (SARS-CoV-2) spread rapidly around the world [1, 2]. Epidemiological studies were established to monitor disease incidence and severity [3–5], understand mechanisms of disease transmission, identify at-risk groups, and guide healthcare responses. Data from adult studies quickly identified that patients on haemodialysis or with a kidney transplant had a greater risk for mortality and morbidity [6–8]. In the UK, these patients were classed as ‘extremely vulnerable’ and were advised to follow special measures, including isolation which impacted work and psychosocial wellbeing [9]. For pediatric patients, COVID-19 registries were also set up, but preliminary assessments of the potential morbidity varied depending on the setting and timepoint [10–12]. The risk of developing severe COVID-19 and concomitant acute kidney injury was unclear early on [13]. Furthermore, pediatric patients were reported to have wider-ranging symptoms compared to the main respiratory symptoms, including diarrhoea and rashes [14].

In the UK, the epidemiology of COVID-19 in adult patients on dialysis and kidney transplantation, hereafter referred to as kidney replacement therapy (KRT), has been studied. The number of cases increased in the first few months of the pandemic, reaching 11% for in-centre dialysis patients, 4% for home dialysis, and 2% for transplant patients, by August 2020 [15]. In pediatric patients, an early study reported five patients with chronic kidney disease up to July 2020, all of whom recovered [13]. Most pediatric studies have so far focused on PCR-positive cases, which target patients who were sufficiently unwell to present to hospital [13]. To study the disease in the whole population, we established the ISpy COVID-19 study to investigate the seroprevalence of anti-SARS-CoV-2 Immunoglobulin G (IgG) in pediatric patients on KRT. Surplus serum samples from two UK nephrology centres were tested for anti-SARS-CoV-2 antibodies using an in-house enzyme-linked immunosorbent assay (ELISA) with population age-matched healthy controls. The serology results complement disease reporting performed by the UK Renal Registry based on positive SARS-CoV-2 antigen testing. We report results from the early developing phase of the COVID-19 pandemic: January 2020 until August 2021.

## Materials and methods

### Population and study setting

ISpy was a longitudinal seroprevalence study conducted in two pediatric nephrology centres in the UK: Nottingham Children’s Hospital and Glasgow Children’s Hospital. Both are large pediatric KRT centres covering a broad geographical area and operate a ‘hub and spoke’ model. The main centre is situated in an urban city, which acts as the ‘hub’ for haemodialysis and transplantation. Post-transplant, patients are followed up for 1 year before being repatriated back to local hospitals with ongoing management from the pediatric nephrologist. Patients may therefore reside in dense urban areas or more sparse rural settings.

### Sampling

Serum samples surplus to routine clinical investigations were obtained from patients on KRT during their hospital outpatient appointments in Nottingham and Glasgow. For patients on in-centre haemodialysis, sera were obtained monthly during clinical review. In Nottingham, patients also performed home monitoring using finger-prick capillary sampling (CountOnMe©). Samples were taken from January 2020 until August 2021 and analysed cross-sectionally for each month.

### SARS-CoV-2 serology

ELISAs were used to evaluate SARS-CoV-2 anti-spike and anti-nucleocapsid protein-specific IgG responses according to optimised methodology, as previously described [16]. All serum samples were first inactivated with 1% Triton X-100 and diluted to 1:600 to meet predefined endpoints for in-house immunoassays. All assays were performed using Biotek Precision liquid handling robots in a class II microbiological safety cabinet. Nunclon assay plates (Nunc) were coated with SARS-CoV-2 antigen at a concentration of 0.5 μg/mL^−1^. For the seroprevalence study, we used a combination of spike S1 subunit (2019-nCoV, His tagged, HEK-293 expressed; Sino Biological) and nucleocapsid (2019-nCoV, His tagged, baculovirus expressed; Sino Biological) antigens. Positive samples were subsequently assessed using individual antigens, with the addition of the full-length spike protein B.1.1.7 variant (HEK-293 expressed; The Native Antigen Company) as it became the dominant strain circulating in the UK during the study period. Gamma chain-specific anti-human IgG horseradish peroxidase (HRP)-conjugate (Sigma A0170-1ML) was used at a 1:30,000 dilution as the detection antibody. Absorbance was measured at 450 nm using a GlowMax Explorer microplate reader (Promega). The cut-off for seropositivity was defined for each assay plate using twice median values from age-matched pre-pandemic pooled negative controls. Antibody binding signals were presented as OD_450_ values divided by the cut-off value to provide an antibody binding ratio for each assay [17]. This permitted separate assays to be compared directly.

### Ethics

Clinical information was obtained from local records by the direct care team. Positive SARS-CoV-2 polymerase chain reaction (PCR) cases were reported to the UK Renal Registry Study and documented in this study. Vaccination dates were obtained locally and cross-referenced with national records. All data were pseudo-anonymized prior to delivery to the research team. This study received regulatory approval on 14 September 2020 (REC reference: 20/HRA/4677).

### Statistics

ISpy was designed as a descriptive seroprevalence study. Additionally, we analysed the correlation between antibody binding signals and clinical characteristics. Comparisons between categorical groups were performed using Kruskal–Wallis tests (accounting for repeated measures) and continuous variables using linear models. We performed statistics using R version 4.1.2. Graphical presentation was done using ggplot2 version 3.3.5.

## Results

### Patient demographics

**Fig. 1:**
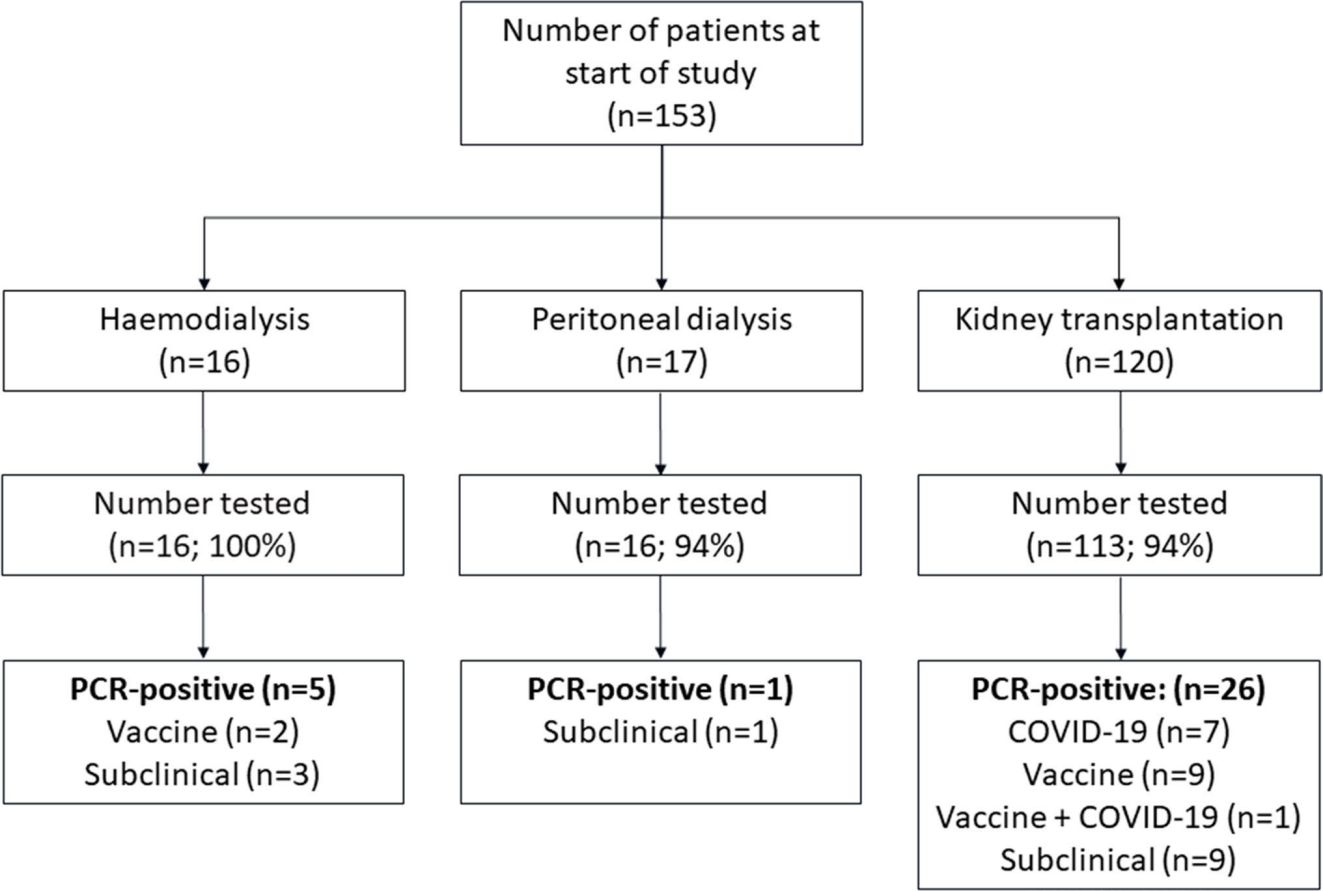
Flow chart of the number (and proportion) of patients tested by RT-PCR for SARS-CoV-2 infection, categorised by kidney replacement therapy modality at the start of the study. Patients were assigned the modality at the time of acquiring a PCR test. Patients testing PCR-positive are further categorised according to the stimulus for the result: SARS-CoV-2 vaccination, symptomatic COVID-19, or subclinical SARS-CoV-2 infection

Samples were available from January 2020 in Nottingham and January 2021 in Glasgow. In total, 520 sera from 145 patients (all 16 patients undergoing in-centre haemodialysis (HD), 16 of 17 patients undergoing peritoneal dialysis (PD), 113 of 120 kidney transplant recipients (Tx)) were analysed (Fig. 1). Demographics at first sampling are presented in Table 1. HD and transplant patients were older (median 11.3 and 12.9 years, respectively) than PD patients (median 5.0 years). Most patients were Caucasian, although among HD patients there were 50% Caucasians and 50% non-Caucasians. In Tx patients, 66% were on prednisolone, and 96% were on calcineurin inhibitors (CNIs; cyclosporine or tacrolimus); 42% were on azathioprine, and 46% were on mycophenolate mofetil (MMF). The median number of tests per patient was 3 (Supplementary Fig. 1). As expected, HD patients had the most tests (median [IQR]: 4.5 [3–6], 1.5 [1–3], and 3 [2–5] for HD, PD, and Tx, respectively). The number of samples from Tx patients varied, reflecting the different follow-up stages post-transplant.

**Table 1:** Clinical characteristics of patients at the start of the study

Characteristic	HD (*n* = 16)	PD (*n* = 16)	Tx (*n* = 113)
Age (at first test, years)	11.3 (8.4–15.4)	5.0 (1.9–13.0)	12.9 (8.8–15.1)
Sex	9/7	10/6	76/37
Male/female	(56%/44%)	(62%/38%)	(67%/33%)
Ethnicity	8/8	13/3	96/17
Caucasian/Non-Caucasian	(50%/50%)	(81%/19%)	(85%/15%)
Primary diagnosis*			
Tubulo-interstitial disease	5 (31%)	6 (38%)	44 (39%)
Glomerulopathy	7 (44%)	5 (31%)	27 (24%)
Familial disease	0	2 (12%)	13 (12%)
Other	4 (25%)	3 (19%)	29 (26%)
Immunosuppression			
Prednisolone			75 (66%)
Calcineurin inhibitors (CNI)			109 (96%)
Azathioprine (Aza)			48 (42%)
Mycophenolate mofetil (MMF)			52 (46%)
Combinations			
Prednisolone/CNI/MMF			20 (18%)
Prednisolone/CNI/Aza			43 (38%)
CNI/MMF			30 (26%)
Other			20 (18%)

^*^Primary diagnoses were categorised using the European Research Association coding system [18]

Results are presented as median (inter-quartile range) and number (percentage)

*HD*, in-centre haemodialysis; *P*D, peritoneal dialysis; *Tx*, kidney transplantation

### Seroprevalence of anti-SARS-CoV-2 antibodies

**Fig. 2:**
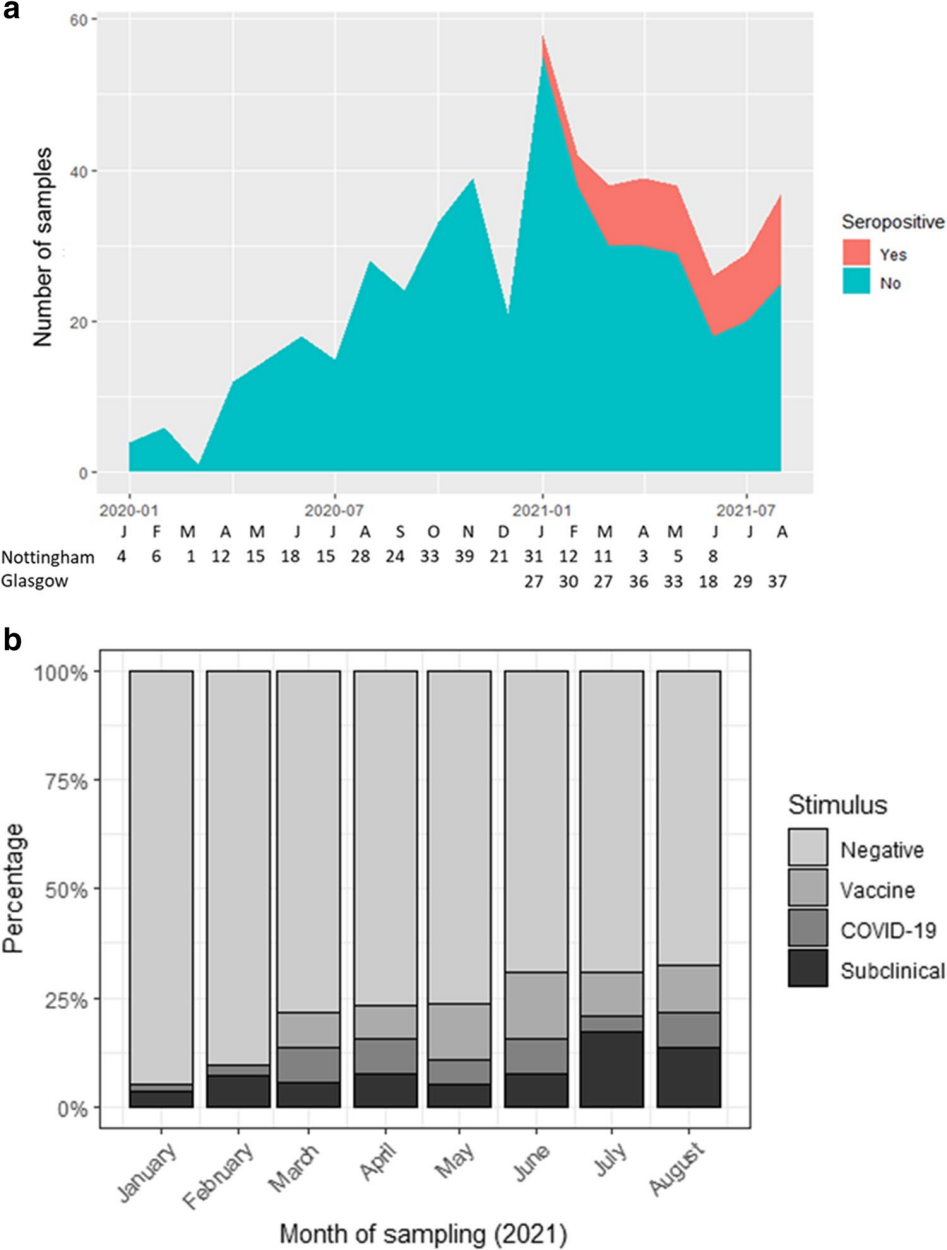
**a** SARS-CoV-2 serological testing during the study period in Nottingham Children’s Hospital and Royal Children’s Hospital, Glasgow. Sample collection month is represented by the first letter with the number of samples collected per month for each centre below. **b** Proportion of samples that were determined to be positive for combined spike subunit 1 and nucleocapsid SARS-CoV-2 antibody binding signal. Antibody-positive results are further categorised from light to dark grey, based on prior exposure (stimulus), including immunisation (vaccine), symptomatic COVID-19, and subclinical SARS-CoV-2 infection, respectively

The number of samples tested each month increased to a maximum of 58 samples in January 2021 (Fig. 2a). Before January 2021, with only Nottingham patients being tested, no samples were found to have an anti-SARS-CoV-2 antibody binding signal above the positive cut-off value. The proportion of seropositive cases gradually increased from 5% in January 2021 to 32% in August 2021 (Fig. 2b). The increase in seropositive cases was coincident with the emergence of the Alpha (B.1.1.7) SARS-CoV-2 variant. More SARS-CoV-2 PCR-positive samples were obtained from Glasgow, reflecting the higher number of samples tested from Glasgow Children’s Hospital (Supplementary Fig. 2). By the end of the study, 32/145 (22%) of patients were PCR-positive (5/16 (31%) HD, 1/16 (6%) PD, and 26/113 (23%) Tx), although the percentage prevalence for PD and Tx patients are likely under-representative due to lower sampling rates (Fig. 1). Among the seropositive patients, 8/32 (25%) had confirmed SARS-CoV-2 by PCR, detected prior to serological testing. Antibodies were detected between 0.8 to 6 months after the positive PCR result. There were three further SARS-CoV-2 PCR-positive patients (all Tx patients) who were negative on subsequent serology samples taken 4–6 months post-exposure.

Vaccination of young adults (16 years and older) in the UK started in February 2021. Twelve patients (2 HD, 10 Tx) received the Pfizer/BioNTech BNT162b2 COVID-19 mRNA vaccine, including ten patients with two doses and two patients with a single dose. Three patients did not show seroconversion after the first dose (all had kidney transplants), but all patients seroconverted after two doses (Supplementary Fig. 3a). Antibodies were still detectable 5.5 months post-vaccination. One of the vaccinees experienced SARS-CoV-2 infection after their first dose of vaccine and so is also grouped as one of the eight SARS-CoV-2 PCR-positive patients referred to above. Serology testing therefore revealed 13/32 (41%) antibody-positive patients without previous known exposure to either SARS-CoV-2 infection or vaccine, representing cases of subclinical infection. Notably, in three of these patients (one HD, two Tx), detectable antibody binding signals were transient and met the positive cut-off value only once, with positivity lasting 1–2 months (data not shown).

### Evaluation of circulating antibody binding signals in SARS-CoV-2 infections

**Fig. 3:**
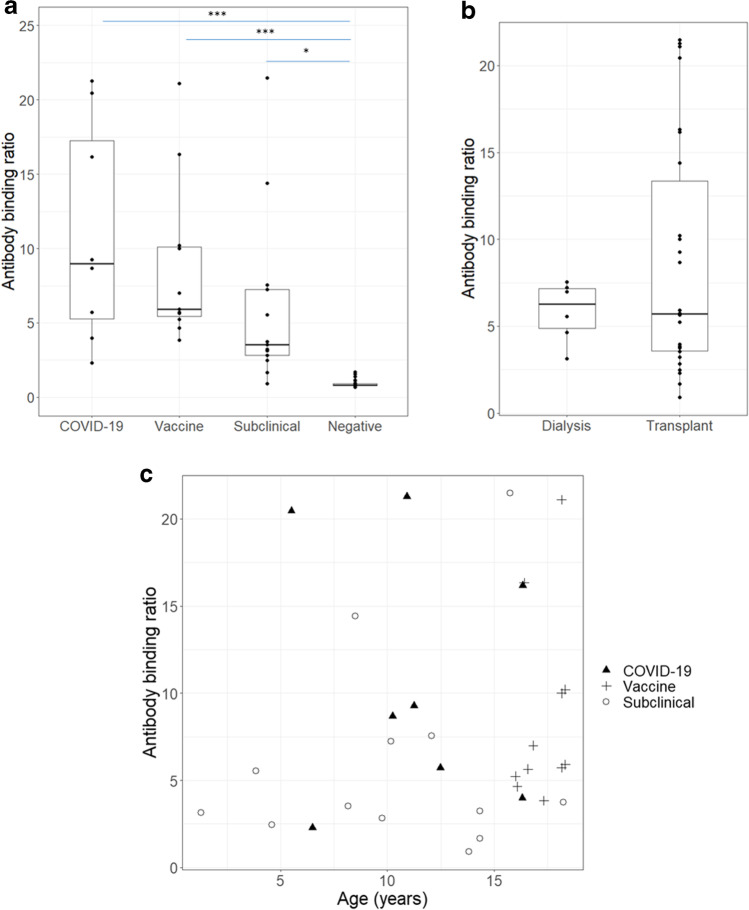
**a** Box plot for SARS-CoV-2 antibody binding signal based on the nature of infection, or vaccine status at the time of positive serological test result. **b** Box plot for SARS-CoV-2 antibody binding signal based on patients’ mode of kidney replacement therapy at the time of positive serological test result. **c** Correlation between antibody binding signal and age at testing (*p* = 0.36). Results are categorised according to the stimulus for the result: SARS-CoV-2 vaccination, COVID-19 or subclinical SARS-CoV-2 infection. Groups were compared using a Kruskal–Wallis test, correcting for multiple comparisons.***p < 0.001, *p < 0.05. HD, in-centre haemodialysis; PD, peritoneal dialysis; Tx, transplantation

We used the data generated for maximum antibody binding signal from each patient to analyse clinical factors associated with antibody response levels (Fig. 3). Overall, the signals generated in antibody binding assays were low relative to negative control sera (median binding ratio 0.8; [IQR 0.5–1.4]). Observed antibody binding ratios were similar regardless of whether the antibody response was generated to natural infection or immunisation, although individuals with symptomatic disease had significantly higher binding ratios (COVID-19: 1.3 [0.7–2.5]; vaccine: 0.8 [0.8–1.4]; subclinical: 0.5 [0.4–1.0]; *p* = 0.2) (Fig. 3a). The mode of KRT also had no association with antibody binding signal (dialysis: 0.9 [0.7–1.0]; transplant: 0.8 [0.5–1.9], *p* = 0.6) (Fig. 3b). There was no correlation between age and observed antibody binding signal; young children developed antibody responses to the viral antigens similarly to older individuals (Fig. 3c). There were insufficient data to determine whether there were associations with immunosuppressive therapies.

### Specific antibody binding to individual SARS-CoV-2 antigens

**Fig. 4:**
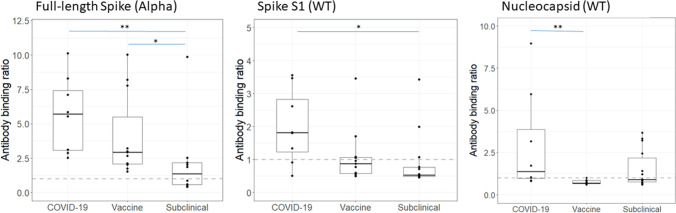
Reactivity of seropositive samples to the full-length spike protein representing the Alpha variant; the S1 subunit of spike protein for the Wuhan wild-type variant (WT) and the nucleocapsid protein of WT. Data is presented as binding ratio of signal: cut-off; dashed lines indicate a ratio of 1. Groups were compared using a Kruskal–Wallis test, correcting for multiple comparisons. ** *p* < 0.01; * *p* < 0.05

We tested seropositive samples for reactivity against separate SARS-CoV-2 antigens: nucleocapsid protein (Wuhan-strain), spike subunit 1 (Wuhan-strain), and full-length spike protein (Alpha-variant) (Fig. 4 and Supplementary Fig. 3). As expected, all patients who were vaccinated were negative for antibodies directed to nucleocapsid antigen (the BNT162b2 vaccine was constructed using spike protein mRNA), except one patient who had PCR-confirmed SARS-CoV-2 infection after vaccination. For patients with PCR-confirmed SARS-CoV-2 exposure, antibody responses were more robust against the full-length spike protein (7/7, 100%) compared to nucleocapsid (4/7, 57%) and S1 (5/7, 71%) (Fig. 4). Vaccination triggered more potent antibody responses towards the full-length spike protein (12/12, 100%) versus the spike S1 subunit (9/12, 75%) in the assays used. Vaccine antibody responses remained elevated for 4–6 months (Supplementary Fig. 3a). Antibody responses peaked 2–3 months after exposure but could be detected up to 15 months later in one patient. In subclinical cases, 6/13 (46%) were positive against nucleocapsid, 3/13 (23%) were positive against S1, and 6/13 (46%) against full-length spike protein. Overall, pediatric patients on KRT developed antibodies which targeted multiple SARS-CoV-2 proteins.

## Discussion

We performed opportunistic population screening of UK pediatric patients on dialysis and post-kidney transplantation at multiple timepoints from January 2020 until August 2021. The seroprevalence of COVID-19 was low in 2020 and increased in 2021 following the emergence of the SARS-CoV-2 Alpha variant and the start of the vaccination programme. A large proportion of seropositive patients (13/32, 41%) had no known prior exposure and might have been missed if using antigen testing and reverse-transcription PCR (RT-PCR) alone.

The UK went into lockdown on 23 March 2020 [19]. Patients on KRT, including pediatric patients, were classified as extremely vulnerable and strongly advised to ‘shield’ and self-isolate. Parents were also advised to work remotely, though this was not enforced through law. Schools were closed until the start of the academic term in September 2020, though anecdotally, patients continued home-schooling. The UK Renal Registry reported confirmed PCR-positive cases (symptomatic testing only), including 10 cases up until 1 September 2020 and a further 36 total cases to 29 December 2020 [20]. As there were 1051 patients < 18 years old on KRT on 31 December 2019, the incidence of COVID-19 in 2020 was low [21]. During this time, our study did not identify any additional cases. Of note, there were two PCR-positive cases from Nottingham Children’s Hospital; the first was determined to lack positive anti-SARS-CoV-2 antibody binding signal, and the second did not have serum samples available post-PCR. There were also three PCR-positive cases from Royal Children’s Hospital, Glasgow in 2020, which only started testing samples in January 2021. As the Alpha variant became dominant in the UK at the start of 2021, the number of COVID-19 cases increased. An additional 107 cases were reported by the UK Renal Registry by 25 August 2021 [20], nine of whom were included in this study. Our serology study therefore detected an additional 13 cases in the two areas.

The seroprevalence in the HD patient population was also low, despite regular sampling, compared to other studies [22]. Seropositive patients were either asymptomatic or vaccinated. Guidelines advised against routine surveillance antigen testing in pediatric HD patients. Patients and staff were screened based on presentation of symptoms and were isolated in cubicles during the dialysis session if PCR-positive. Infection control measures were therefore effective, and transmission between children is likely lower, as evidenced by other studies in the literature [23, 24].

The gold standard for diagnosing SARS-CoV-2 infection is RT-PCR testing to detect viral RNA, and sensitivity is highest at 7–10 days after exposure. Outside this time frame, the false negative rate can range between 38 and 66% [25, 26]. Studies assessing the diagnostic accuracy of ELISA antibody tests are often performed in in-patient hospital settings and based on patients with positive PCR tests. As expected, antibody detection peaks after 14 days for IgG, although IgM and IgA antibodies are typically detected earlier [27, 28]. The sensitivity of the pooled ELISA tests was 84.3% (95% confidence interval; 75.6% to 90.9%) [28]. Antibodies were shown to persist between 5 and 7 months after infection, regardless of disease severity [29]. The predictive performance of antibody tests also changes with disease prevalence. Therefore, in the setting of surveillance screening of asymptomatic cases (low prior probability), the negative predictive value is higher, while the positive predictive value decreases. The antibody detection assay used in this study was calibrated using age-matched negative controls to reduce the likelihood of a false positive result.

Our results in 2020 are consistent with other population seroprevalence studies which showed low positive rates in the early pandemic period [29]. Morello et al. performed a stratified sampling study in a heterogeneous patient group (patients on KRT and/or immunosuppressed) in Italy from 15 July to 14 September 2020, finding a 3/178 (2%) positive serology rate [30]. In a more targeted approach using PCR testing of symptomatic Tx patients and hospital screening, the positivity rate was 4.4% in the USA from April to September 2020, with a third of patients being asymptomatic [12]. Compared to the general population of children in the UK (5–18 years old), the point prevalence using PCR testing was as low as 0.1% during May 2020, increased steadily after September 2020, and reached 2% in January 2021 [31]. The low detection rate in our study therefore followed national trends in the patients’ peers.

From January 2021, more patients tested positive for anti-SARS-CoV-2 antibodies; 41% of seropositive patients had no known prior exposure. There is now good evidence that children have a low risk of developing severe COVID-19 [32–34]. Our seroprevalence data from asymptomatic or subclinical patients adds to this evidence. Studies in other cohorts of immunosuppressed children with oncological and rheumatological conditions have also shown a low risk of morbidity and mortality [35, 36].

Given that several individuals in this study generated antibodies that provided binding signal only just above the strictly defined threshold level for positivity, it is plausible that our results are an underestimate of all true antibody-positive patients in the community. Moreover, antibody binding ratios were generally low across our cohort, when calculated relative to age-matched negative controls and compared to immunised adults [17]. This may be unsurprising given that children may be less likely to seroconvert than adults when SARS-CoV-2 infection is mild, irrespective of viral load [37]. For pediatric SARS-CoV-2 infection, a lower magnitude of anti-nucleocapsid IgG antibody response was also observed compared to adult infections [38]. This may partly explain the low seropositivity rate in our cohort, as the primary assay used detected both anti-nucleocapsid and anti-spike S1 IgG antibodies, whereas full-length spike was used for confirmation of positive results [39].

We also quantified the degree of antibody binding above the cut-off threshold, although caution should be taken when interpreting the data owing to limited patient numbers. Antibody binding signal was lower (though not statistically significant) in patients with subclinical infection, likely due to reduced viral load or effective viral clearance, which would limit B cell activation. The lower antibody binding signal observed in dialysis patients may be attributed to the high proportion of subclinical cases. Our study also found high antibody binding signals among our cohort of transplant patients compared to transplant patients in other studies. This may be related to lower immunosuppression and lower rates of mycophenolate mofetil compared to other studies [40–42]. Notably, we did not measure viral neutralisation or T-cell responses in post-vaccinated samples [42]. The reduced severity of COVID-19 disease in children has been shown to be due to more effective viral clearance in respiratory epithelia, which leads to a dampened antibody response [43]. Mortality in adult HD patients relates to both the pro-inflammatory state of dialysis as well as reduced immune competence from uraemia. The myriad local and systemic, innate and adaptive responses therefore likely explain the reduced risk of severe disease in children, even where immunosuppressed.

We acknowledge some limitations in our study. ISpy was conducted in two pediatric KRT centres in the UK and may not be representative of the national pattern of infection. In particular, the spread of infection and peak incidence varied geographically, although the whole of the UK eventually followed similar patterns. Though this was a longitudinal study over a long timeframe, sampling was opportunistic, so the majority of, but not all, patients had multiple samples. The low positive rate in patients on PD could represent the lower frequency of sampling. Transplant patients were typically repatriated to their local hospitals after 1-year post-transplant and had no samples in the KRT centre. However, in Nottingham, samples were retrieved from patients who performed home testing and remote follow-up appointments and could therefore assess community transmission. This study was also not designed to assess vaccine antibody responses as the availability of vaccines was not known at the time of study design.

## Conclusions

The seroprevalence of anti-SARS-CoV-2 in pediatric patients on dialysis and kidney transplantation in the UK was low in the first year of the pandemic and increased in the first half of 2021. Population infection control methods were likely effective at preventing transmission, though this needs to be balanced against the unintended mental health consequences of social isolation. Serological surveillance complements studies using RT-PCR or antigen testing to build a more complete picture of the epidemiology of COVID-19, particularly in identifying subclinical cases, and is therefore important to guide public health responses.

## Supplementary Information

Below is the link to the electronic supplementary material.

Graphical Abstract (PPTX 593 KB)

Supplementary file1 (DOCX 360 KB)

## Data Availability

The datasets generated during and/or analysed during the current study are available from the corresponding author on reasonable request.
