# Supplementary material for: Increasing SARS-CoV-2 seroprevalence among UK pediatric patients on dialysis and kidney transplantation between January 2020 and August 2021
Source: Pediatr Nephrol. 2023 Jun 1;38(11):3745–55. doi: 10.1007/s00467-023-05983-1 (PMC10233184; doi:10.1007/s00467-023-05983-1)
Supplement: Supplementary file 1 — Graphical Abstract (PPTX 593 KB) [file 467_2023_5983_MOESM1_ESM.pptx]

## Slide 1
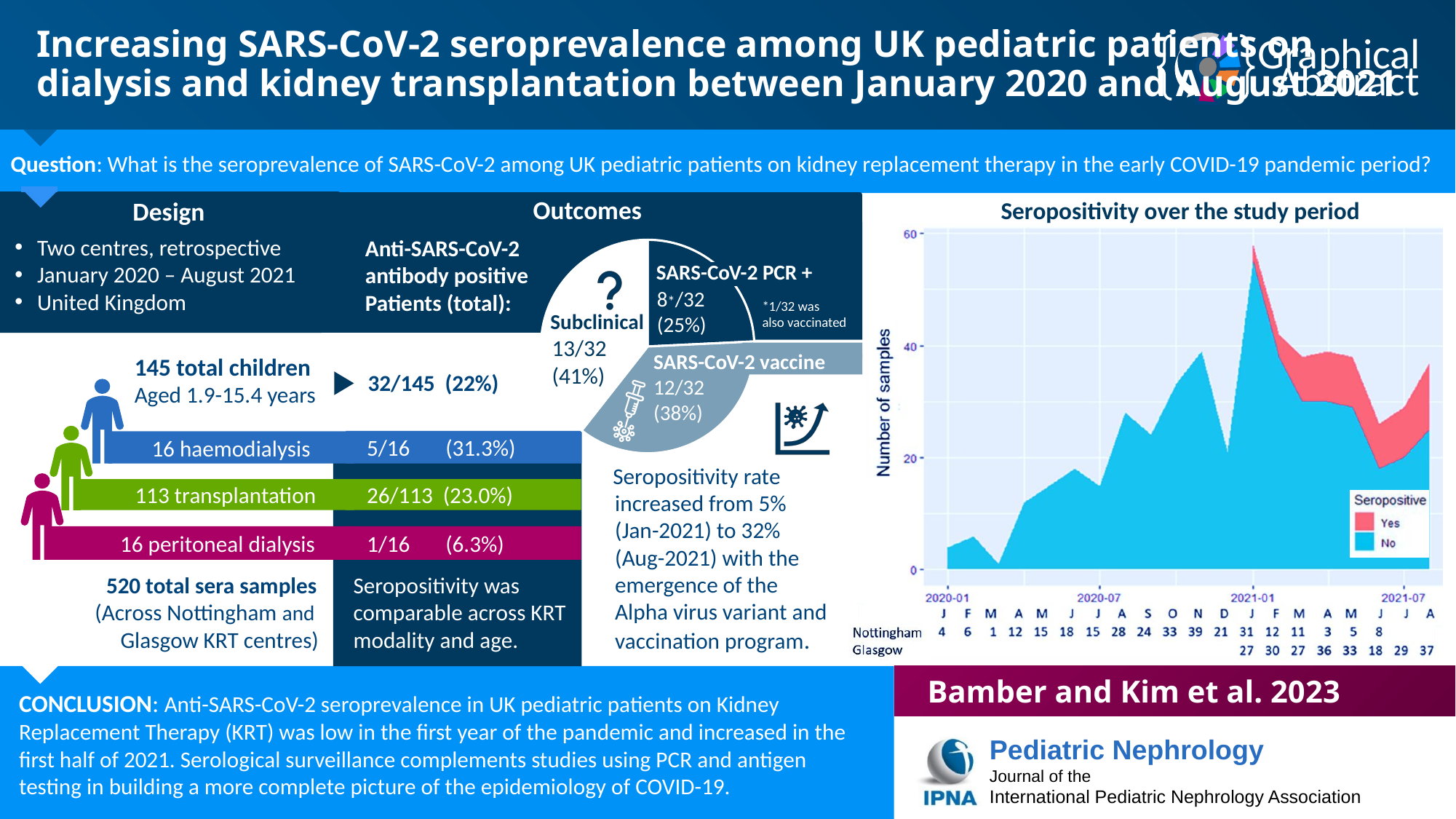

Increasing SARS-CoV-2 seroprevalence among UK pediatric patients on
dialysis and kidney transplantation between January 2020 and August 2021
Question: What is the seroprevalence of SARS-CoV-2 among UK pediatric patients on kidney replacement therapy in the early COVID-19 pandemic period?
Design
Outcomes
 Seropositivity over the study period
Anti-SARS-CoV-2
antibody positive
Patients (total):
### Chart
| Category | Column2 |
|---|---|
| SARS-CoV-2 | 8.0 |
| SARS-CoV-2 Vaccine | 12.0 |
| Unkown stimulus | 13.0 |
Two centres, retrospective
January 2020 – August 2021
United Kingdom
SARS-CoV-2 PCR +
8*/32
(25%)
Subclinical
SARS-CoV-2 vaccine
12/32
(38%)
13/32 (41%)
*1/32 was
also vaccinated
 32/145 (22%)
145 total children
Aged 1.9-15.4 years
 16 haemodialysis
 113 transplantation
 16 peritoneal dialysis
 5/16 (31.3%)
increased from 5% (Jan-2021) to 32% (Aug-2021) with the emergence of the Alpha virus variant and vaccination program.
Seropositivity rate
 26/113 (23.0%)
 1/16 (6.3%)
Seropositivity was comparable across KRT modality and age.
 520 total sera samples
 (Across Nottingham and
 Glasgow KRT centres)
Bamber and Kim et al. 2023
CONCLUSION: Anti-SARS-CoV-2 seroprevalence in UK pediatric patients on Kidney Replacement Therapy (KRT) was low in the first year of the pandemic and increased in the first half of 2021. Serological surveillance complements studies using PCR and antigen testing in building a more complete picture of the epidemiology of COVID-19.
