# Supplementary material for: Increasing SARS-CoV-2 seroprevalence among UK pediatric patients on dialysis and kidney transplantation between January 2020 and August 2021
Source: Pediatr Nephrol. 2023 Jun 1;38(11):3745–55. doi: 10.1007/s00467-023-05983-1 (PMC10233184; doi:10.1007/s00467-023-05983-1)
Supplement: Supplementary file 2 — Supplementary file1 (DOCX 360 KB) [file 467_2023_5983_MOESM2_ESM.docx]

**Increasing SARS-CoV-2 seroprevalence among UK pediatric patients on dialysis and kidney transplantation between January 2020 and August 2021**

**Holly N. Bamber^1¶^, Jon Jin Kim^2,3¶^, Ben C. Reynolds^4^, Javairiya Afzaal^2^, Andrew J. Lunn^2^, Patrick J. Tighe^1^, William L. Irving^1,5,6^, Alexander W. Tarr^1,5,6*^**

^1^School of Life Sciences, University of Nottingham, UK

^2^Department of Paediatric Nephrology, Nottingham University Hospitals, UK

^3^Centre for Kidney Research and Innovation, University of Nottingham, UK

^4^Department of Paediatric Nephrology, Royal Hospital for Children, Glasgow, UK

^5^NIHR Nottingham Biomedical Research Centre, Nottingham University Hospitals NHS Trust and the University of Nottingham, Nottingham, UK

^6^Wolfson Centre for Global Virus Research, The University of Nottingham, Nottingham, UK

*Correspondence to: Alexander W. Tarr, Microbiology, Queen's Medical Centre,

Nottingham, NG7 2UH, UK. Email: alex.tarr@nottingham.ac.uk. Tel: 0115 74 86468.

^¶^These authors contributed equally to this work.

**Supplementary Figures**

**Supplementary Figure 1a**

**
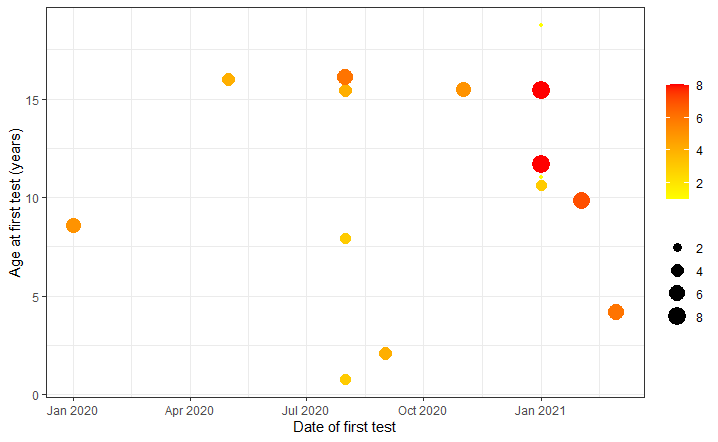
**

**Supplementary Figure 1b**

**
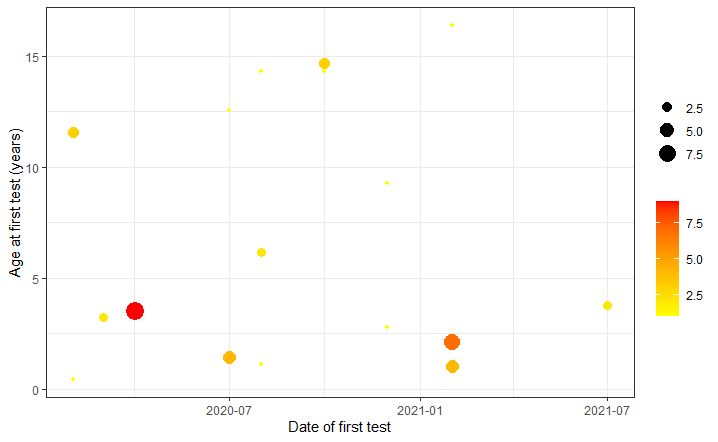
**

**Supplementary Figure 1c**

**
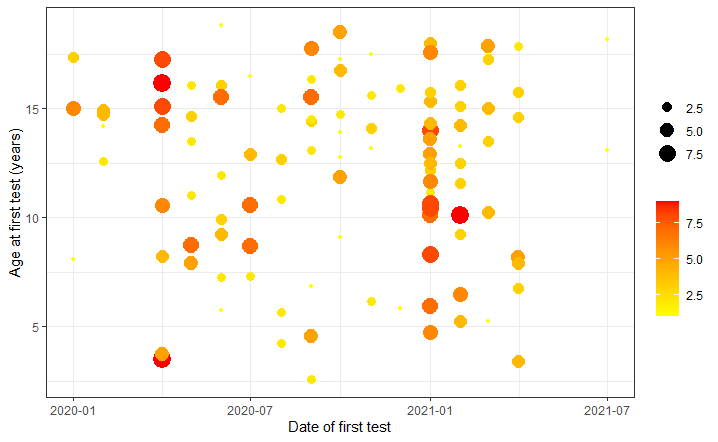
**

**Supplementary Figure 1** Number of tests for each patient based on date and age at the first sampling time point for: (a) in-centre haemodialysis, (b) peritoneal dialysis, and (c) kidney transplantation. Both the size of the point and the colour intensity represent the number of tests performed for that patient.

**Supplementary Figure 2**

**
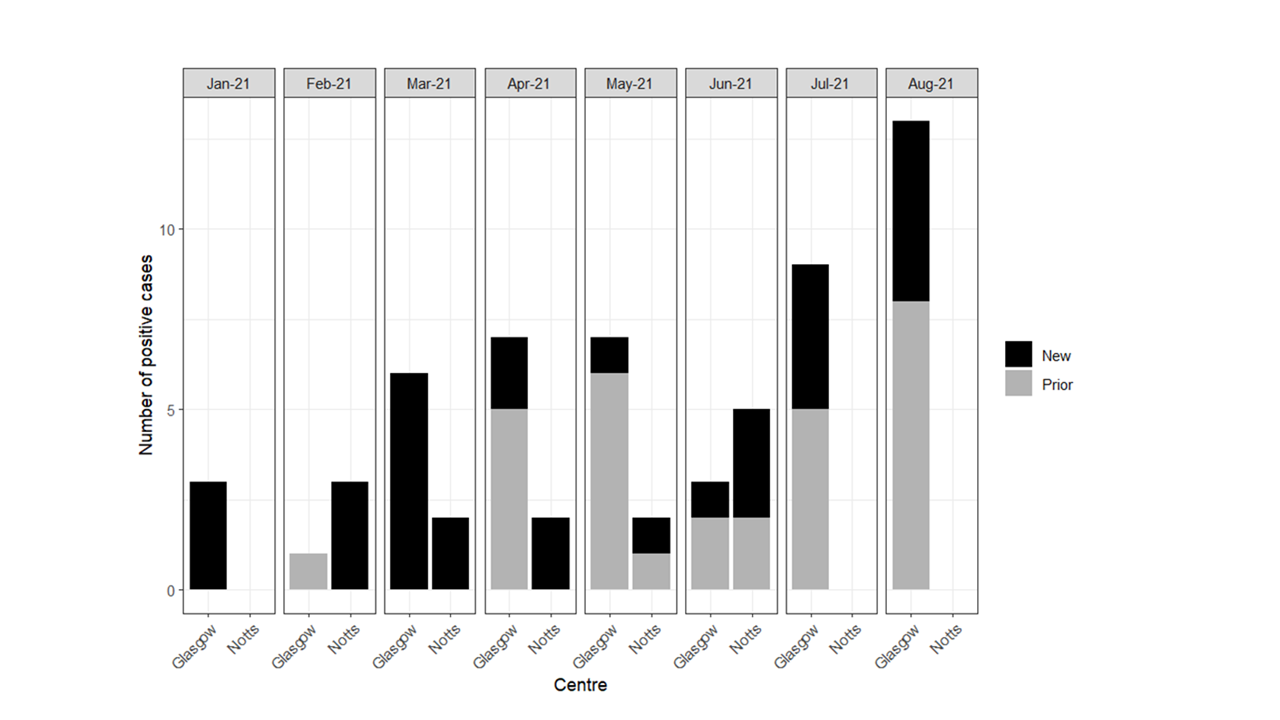
**

**Supplementary Figure 2** Number of seropositive cases per centre: Glasgow and Nottingham (Notts). Black bars represent novel SARS-CoV-2 infections; grey bars represent patients who had prior detected anti-SARS-CoV-2 antibodies.

**Supplementary Figure 3a**


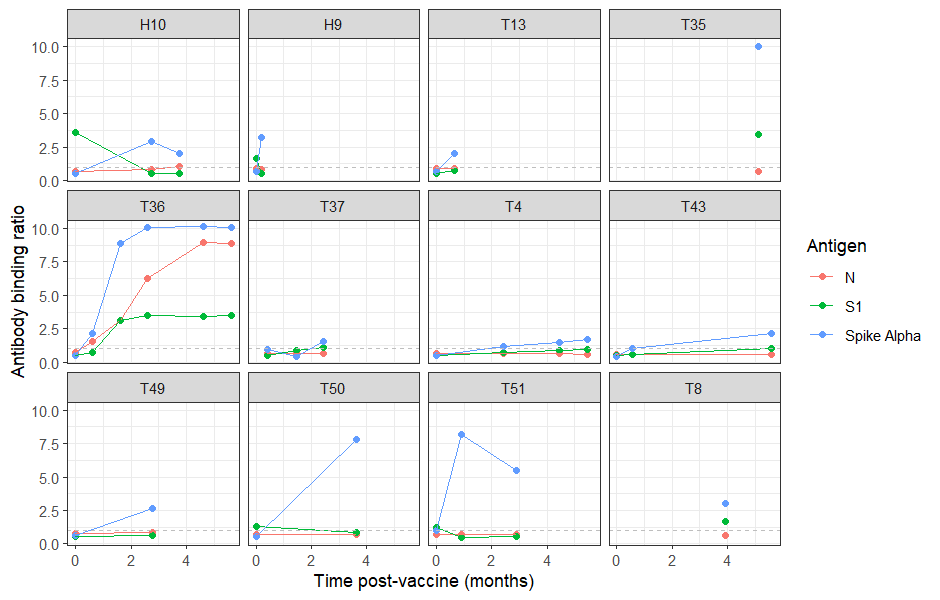


**Supplementary Figure 3b**


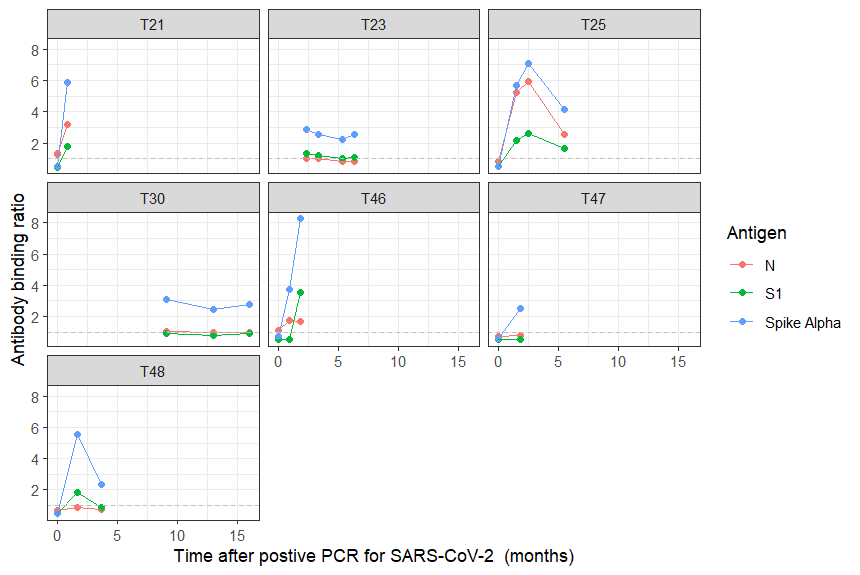


**Supplementary Figure 3** Antibody binding signal for specific SARS-CoV-2 proteins in patients post vaccination (a) and positive PCR test result (b). Patient T36 experienced SARS-CoV-2 infection following their first vaccination. Longitudinal samples were analysed from excess sera from outpatient appointments or haemodialysis blood tests. The most recent sample before the positive test (where available) was taken as the baseline. H: haemodialysis, T: transplant, N: nucleocapsid (Wuhan wild-type variant), S1: spike subunit 1 (Wuhan wild wild-type variant), spike Alpha (Full-length spike protein; Alpha variant). Values are presented as a ratio of signal to cut off; dashed lines indicate a value of 1.
